# Case Report: A Very Low Birth Weight Female Infant With Congenital Bilateral Periventricular Leukomalacia, Born to a Mother With Coronavirus Disease 2019

**DOI:** 10.3389/fped.2022.887132

**Published:** 2022-05-09

**Authors:** Keisuke Kobata, Nanae Yutaka, Hiroshi Ogasawara, Aya Mima, Kaho Suzuki, Ryoichi Hazama, Ran D. Goldman, Makoto Nabetani

**Affiliations:** ^1^Department of Pediatrics, Yodogawa Christian Hospital, Osaka, Japan; ^2^Department of Obstetrics and Gynecology, Yodogawa Christian Hospital, Osaka, Japan; ^3^Department of Pediatrics, University of British Columbia, Vancouver, BC, Canada

**Keywords:** COVID-19, very low birth weight baby, periventricular leukomalacia, PVL, ultrasound, MRI

## Abstract

A 26-year-old primipara woman with COVID-19 performed an emergency Cesarean section due to further hypoxemia at 28 weeks 5/7 days gestation. The female neonate was born weighing 1,347 gram with an Apgar score of four at 1 min, three at 5 min, and eight at 10 min. RT-PCR from nasopharyngeal swabs for COVID-19 were performed at birth, 24 h, and 48 h after birth, all of which were negative. On head ultrasound bilateral cystic lesions were found in the anterior horn of the lateral ventricles at birth. A brain magnetic resonance imaging (MRI) test at 56 days of life (corrected 36 weeks and 6/7 days) revealed cystic lesions with T1 low signal, T2 high signal, and T2 Flair high signal around the anterior horn of the lateral ventricle and We diagnose it as Grade 2 periventricular leukomalacia (PVL). She was discharged on day 64 of life, with no abnormality on exam. While the majority of neonates born to women with COVID-19 during pregnancy have favorable outcome, we report a case of a neonate with Grade 2 periventricular leukomalacia and this should prompt clinicians to monitor fetal cerebral function and structure shortly after birth.

## Introduction

Coronavirus disease 2019 (COVID-19) affected millions of people worldwide since early 2020 (1) including pregnant women. There is limited reporting of the clinical characteristics and outcomes of neonates, especially premature newborns or those infected during pregnancy. We report a very low birth weight female infant with congenital bilateral periventricular cystic lesions born to a mother with COVID-19.

## Case Presentation

**FIGURE 1:**
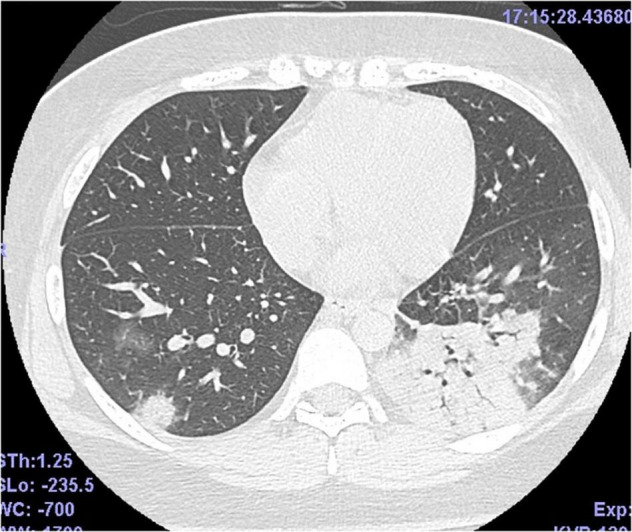
Thoracic CT scan of the mother. Bilateral ground glass opacities and consolidation.

**FIGURE 2:**
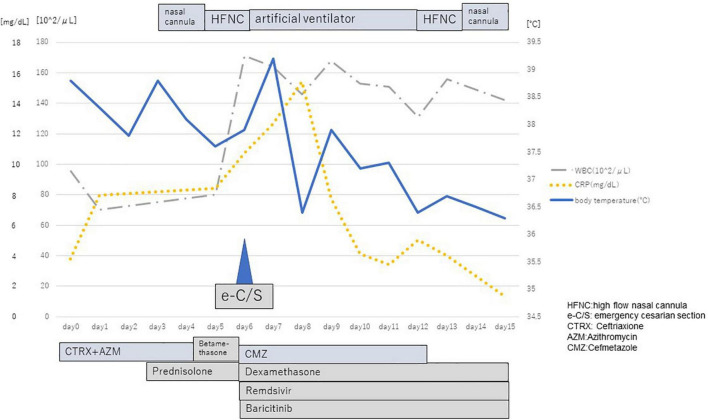
Chart of clinical condition of the patient’s mother.

A 26-year-old primipara woman reported fever and headache at 27 weeks 4/7 days gestation. She was diagnosed with COVID-19 using real-time reverse transcription polymerase chain reaction (rRT-PCR:GeneXpert^®^, Beckman Coulter Inc., United States) from a sample done via nasopharyngeal swab two days later (The threshold cycle was 20.5), and was admitted to the hospital. She didn’t diagnose premature rupture of the membrane and had no medical history, except for obesity. Treatment on day four of admission included oxygen with nasal cannula and her clinical condition deteriorated. A thoracic computed tomography (CT) scan revealed bilateral ground glass opacities and consolidation (Figure 1). With progressive hypoxemia she was placed on high flow nasal cannula (HFNC) and was given intramuscular corticosteroids to facilitate fetal pulmonary maturation. The next day her providers performed an emergency Cesarean section due to further hypoxemia (shown in Figure 2).

**FIGURE 3:**
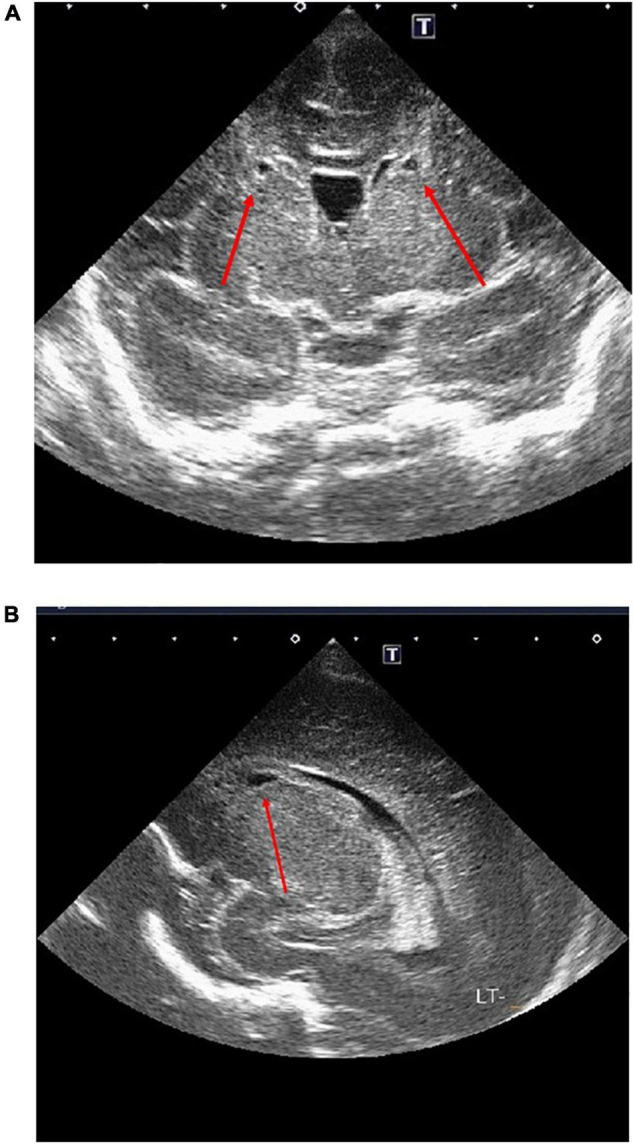
**(A,B)** Head ultrasound. Day 0 Red allows show bilateral cystic lesions by the anterior horn of the each ventricles.

**FIGURE 4:**
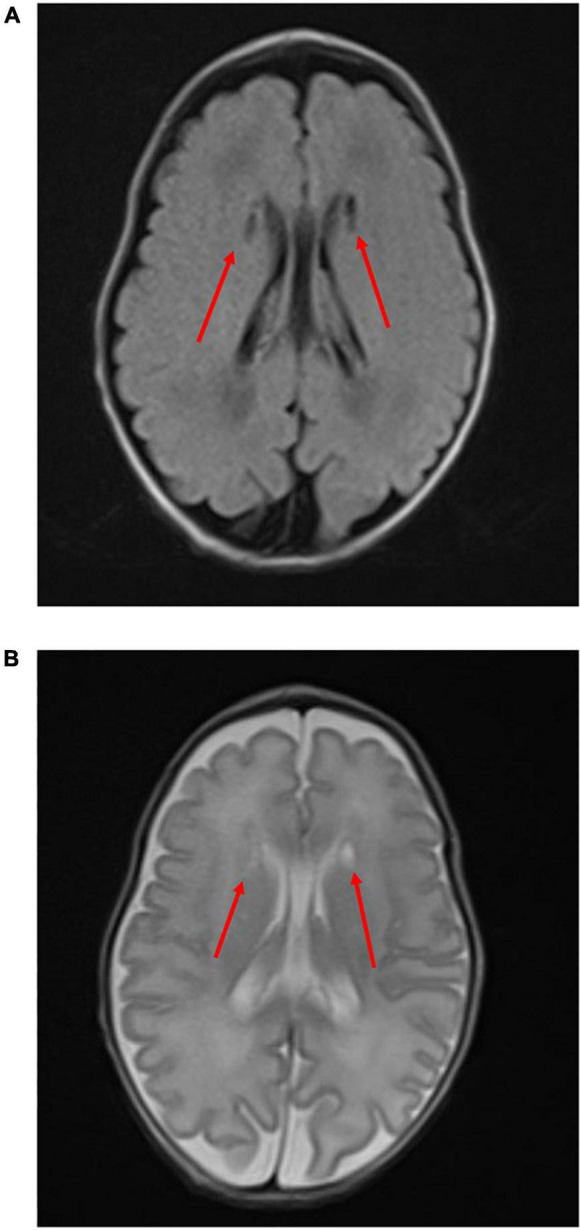
Brain MRI. **(A)** T2 flair: Red allows show bilateral cystic lesions. **(B)** T2-weighted: Red allows show bilateral cystic lesions.

**FIGURE 5:**
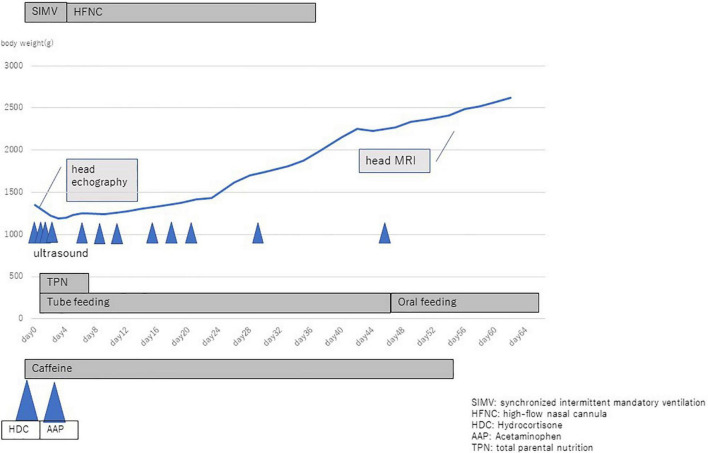
Chart of clinical condition of the patient.

The female neonate was born weighing 1,347 gram with an Apgar score of four at 1 min, three at 5 min, and eight at 10 min. Upon birth the baby was diagnosed with neonatal idiopathic respiratory distress syndrome (RDS) based on increased oxygen demand and a chest X-ray, and immediately thereafter surfactant was administered intra-tracheally. rRT-PCR from nasopharyngeal swabs for COVID-19 were performed at birth, 24 h, and 48 h after birth, all of which were negative. Toxoplasma gondii, rubella, cytomegalovirus (CMV), and herpes simplex virus (HSV) testing (TORCH) were negative. On head ultrasound bilateral cystic lesions were found in the anterior horn of the lateral ventricles at birth and 4 days of life (Figures 3A,B). A brain magnetic resonance imaging (MRI) test at 56 days of life (corrected 36 weeks and 6/7 days) revealed cystic lesions with T1 low signal, T2 high signal, and T2 Flair high signal around the anterior horn of the lateral ventricle (Figures 4A,B). The diagnosis was Grade 2 periventricular leukomalacia (PVL) (based on the definition (2) of the presence of punctate lesions <= 3 mm in individual size in periventricular white matter on either or both of the T1/T2-weighted images and the presence of lesions in bilateral corticospinal tracts or, more extensively, with >= 3 lesions per hemisphere). Differential diagnosis includes connatal cyst and subependymal pseudocyst. We diagnosed PVL in the viewpoint of location, form, size and number of cysts (3). On admission, the newborn needed intermittent mechanical ventilation for four days and high-flow nasal cannula for 32 days. She was discharged on day 64 of life, with no abnormality on exam (shown in Figure 5). She is now 6 months old and even acquired the ability to turn over. We are planning to follow up the baby until her school age, because there have been some reports that individuals with PVL show symptom of cognitive impairment (4).

## Discussion

Periventricular leukomalacia was first described by Banker in 1962 (5). Now PVL is one of most frequent disease which could lead to cerebral palsy in preterm newborns. The incidence of cystic PVL in very low birth weight (VLBW) neonates in Japan during 2003–2012 was 3.3%.^1^ The neuropathologic hallmarks of PVL are microglial activation and focal and diffuse periventricular depletion of premyelinating oligodendroglia. Premyelinating oligodendroglia are highly vulnerable to death caused by glutamate, free radicals, and proinflammatory cytokines (6). We report a unique case of a VLBW female neonate with congenital bilateral PVL, born to a COVID-19 positive mother.

A multinational study involving 18 countries (including Japan) early in the pandemic revealed that pregnant individuals with COVID-19 infection had substantial increase in severe maternal morbidity and maternal mortality, as well as neonatal complications, compared to non-infected women (7). Vertical transmission to neonates demonstrated favorable neonatal outcomes. Newborns were asymptomatic or had mild respiratory symptoms with a recovery within two weeks (8).

Several reports described increased rate of preterm birth, up to 22% in a recent systematic review (9). Current available literature does not report increased incidence of neonates that are small for gestational age or an increase in birth defects in the setting of COVID-19 in the second and third trimester of pregnancy, compared with population baseline (10).

Zeng et al. reported that among eight neonates that underwent brain MRI at corrected gestational age of 44 weeks, five were diagnosed with COVID-19 and of those, three had abnormal signal in the white matter and delayed myelination (*n* = 1), delayed myelination and brain dysplasia (*n* = 1), and abnormal signal in bilateral periventricular areas (*n* = 1) (11).

Engert et al. reported a case of severe brain damage after SARS-CoV-2-mediated hyper inflammation during pregnancy. In the report, intracranial inflammation and coagulopathy were documented as well as petechial bleeding (without abnormal platelet count) and highly elevated D-dimer and White Blood Cell (WBC) count (12).

We suggest that the neonate we report had a post-COVID-19 systemic inflammatory response during pregnancy that resulted in pathological fetal circulation, followed by a coagulopathy and preterm labor. It is possible that placental over expression of angiotensin-converting enzyme 2 which can critically modulate the SARS-CoV-2 receptor caused modulated hemodynamics within the utero-placental unit. Secondly, the pro-inflammatory cytokine storm invoked by SARS-CoV-2 may have induce severe inflammation with deleterious consequences on the fetal brain (e.g., development of periventricular leukomalacia) (13).

Favre et al. reported two cases of decreased fetal movements and abnormal fetal heart rhythm five days after mild maternal COVID-19, requiring emergency Cesarean section at 29 weeks and 3/7 days and 32 weeks and 1/7 days of gestation, leading to brain injury. Placental examination revealed extensive and multifocal chronic inter-villositis, with intense cytoplasmic positivity for SARS-CoV-2 spike antibody and SARS-CoV-2 detection by RT-qPCR. Vertical transmission was confirmed in one case, and both neonates developed extensive cystic PVL (13).

## Conclusion

We report a case of a neonate with Grade 2 periventricular leukomalacia and this should prompt clinicians to monitor fetal cerebral function and structure shortly after birth under careful monitoring of cardio respiratory condition during investigation following approved guidelines.

## Data Availability Statement

The datasets for this article are not publicly available due to concerns regarding participant/patient anonymity. Requests to access the datasets should be directed to the corresponding authors.

## Ethics Statement

The studies involving human participants were reviewed and approved by the Institutional Review Board of Yodogawa Christian Hospital. Written informed consent to participate in this study was provided by the participants’ legal guardian/next of kin.

## Author Contributions

KS and RH consulted in mother. KK, NY, HO, and AM consulted in the prenatal period and designed. KK ang MN wrote the draft. KK, NY, and AM provided medical care at NICU. KK participated in the data collection and provided histology analysis. RG reviewed the literature and participated in the publication. All authors contributed to the article and approved the submitted version.

## Conflict of Interest

The authors declare that the research was conducted in the absence of any commercial or financial relationships that could be construed as a potential conflict of interest. The handling editor TM declared a past co-authorship with one of the authors MN.

## Publisher’s Note

All claims expressed in this article are solely those of the authors and do not necessarily represent those of their affiliated organizations, or those of the publisher, the editors and the reviewers. Any product that may be evaluated in this article, or claim that may be made by its manufacturer, is not guaranteed or endorsed by the publisher.

## Footnotes

1
https://plaza.umin.ac.jp/nrndata/syukei.htm
